# RETRACTION: Risk Factors for Surgical Site Infections Following Open Reduction and Internal Fixation in Patients With Tibial Plateau Fractures

**DOI:** 10.1111/iwj.70591

**Published:** 2025-04-21

## Abstract

**RETRACTION:** SunS. , YangG. , ZhangY. , and LiuY. , “Risk Factors for Surgical Site Infections Following Open Reduction and Internal Fixation in Patients With Tibial Plateau Fractures,” International Wound Journal 21, no. 3 (2024): e14496, 10.1111/iwj.14496.37969024 PMC10898405

The above article, published online on 15 November 2023, in Wiley Online Library (http://onlinelibrary.wiley.com/), has been retracted by agreement between the journal Editor in Chief, Professor Keith Harding; and John Wiley & Sons Ltd. Following an investigation by the publisher, all parties have concluded that this article was accepted solely on the basis of a compromised peer review process. In addition, further investigation by the publisher found that the ethical approval information for the study is not consistent between the Materials and Methods and the Ethics Statement. In view of the clear evidence of compromised peer review, the parties agreed that the paper must be retracted. The authors did not respond to our notice regarding the retraction.

**RETRACTION:** S. Sun , G. Yang , Y. Zhang , and Y. Liu , “Risk Factors for Surgical Site Infections Following Open Reduction and Internal Fixation in Patients With Tibial Plateau Fractures,” International Wound Journal 21, no. 3 (2024): e14496, 10.1111/iwj.14496.37969024 PMC10898405

The above article, published online on 15 November 2023, in Wiley Online Library (http://onlinelibrary.wiley.com/), has been retracted by agreement between the journal Editor in Chief, Professor Keith Harding; and John Wiley & Sons Ltd. Following an investigation by the publisher, all parties have concluded that this article was accepted solely on the basis of a compromised peer review process. In addition, further investigation by the publisher found that the ethical approval information for the study is not consistent between the Materials and Methods and the Ethics Statement. In view of the clear evidence of compromised peer review, the parties agreed that the paper must be retracted. The authors did not respond to our notice regarding the retraction.

